# The 14-3-3ζ Protein Binds to the Cell Adhesion Molecule L1, Promotes L1 Phosphorylation by CKII and Influences L1-Dependent Neurite Outgrowth

**DOI:** 10.1371/journal.pone.0013462

**Published:** 2010-10-18

**Authors:** Elisa M. Ramser, Gerrit Wolters, Galina Dityateva, Alexander Dityatev, Melitta Schachner, Thomas Tilling

**Affiliations:** 1 Zentrum für Molekulare Neurobiologie Hamburg, University of Hamburg, Hamburg, Germany; 2 Department of Neuroscience and Brain Technologies, Italian Institute of Technology, Genova, Italy; 3 Keck Center for Collaborative Neuroscience and Department of Cell Biology and Neuroscience, Rutgers University, Piscataway, New Jersey, United States of America; University of Oldenburg, Germany

## Abstract

**Background:**

The cell adhesion molecule L1 is crucial for mammalian nervous system development. L1 acts as a mediator of signaling events through its intracellular domain, which comprises a putative binding site for 14-3-3 proteins. These regulators of diverse cellular processes are abundant in the brain and preferentially expressed by neurons. In this study, we investigated whether L1 interacts with 14-3-3 proteins, how this interaction is mediated, and whether 14-3-3 proteins influence the function of L1.

**Methodology/Principal Findings:**

By immunoprecipitation, we demonstrated that 14-3-3 proteins are associated with L1 in mouse brain. The site of 14-3-3 interaction in the L1 intracellular domain (L1ICD), which was identified by site-directed mutagenesis and direct binding assays, is phosphorylated by casein kinase II (CKII), and CKII phosphorylation of the L1ICD enhances binding of the 14-3-3 zeta isoform (14-3-3ζ). Interestingly, in an *in vitro* phosphorylation assay, 14-3-3ζ promoted CKII-dependent phosphorylation of the L1ICD. Given that L1 phosphorylation by CKII has been implicated in L1-triggered axonal elongation, we investigated the influence of 14-3-3ζ on L1-dependent neurite outgrowth. We found that expression of a mutated form of 14-3-3ζ, which impairs interactions of 14-3-3ζ with its binding partners, stimulated neurite elongation from cultured rat hippocampal neurons, supporting a functional connection between L1 and 14-3-3ζ.

**Conclusions/Significance:**

Our results suggest that 14-3-3ζ, a novel direct binding partner of the L1ICD, promotes L1 phosphorylation by CKII in the central nervous system, and regulates neurite outgrowth, an important biological process triggered by L1.

## Introduction

L1 is a cell adhesion molecule of the immunoglobulin superfamily which is essential for normal development of the mammalian nervous system. Constitutively L1-deficient mice display severe brain malformations, in particular hydrocephalus and agenesis of the corpus callosum [1], [2]. Similar deficits have been discovered in humans carrying mutations in their *L1CAM* gene [3]. It has been demonstrated that cell recognition via L1 is important both for axon outgrowth and for neuronal migration (reviewed in [4], [5]). These processes are likely to require dynamic control of L1-mediated cell adhesion, for instance by internalization of L1, regulating the availability of L1 on the cell surface. In support of this assumption, endocytotic trafficking of L1 has proved to be important for axon elongation [6]. Regulated L1 internalization depends on interactions of its intracellular domain with signaling, cytoskeletal, and adaptor molecules [7]. In particular, the tyrosine-based sorting motif Y^1176^RSL, which interacts with the adaptor protein AP-2, is necessary for clathrin-mediated endocytosis of L1 [8]. Phosphorylation of Y^1176^ by the nonreceptor tyrosine kinase p60src prevents L1 binding to AP-2 [9]. This motif overlaps with the RSLE sequence, encoded by the alternatively spliced exon 28 [10]. The RSLE sequence is present only in L1 from neurons, but not in L1 expressed by non-neuronal cells such as Schwann cells [11]. Ser^1181^, the second serine residue of the YRSLESDNEE sequence in the L1ICD, can be phosphorylated by CKII [12]. This posttranslational modification most probably plays a critical role in endocytotic trafficking and L1-stimulated axon elongation [13]. However, molecular mechanisms by which CKII–mediated phosphorylation could influence L1 function have not been investigated so far. Notably, the resulting RSLEpS sequence is a potential binding motif for 14-3-3 proteins [14], and analysis of transgenic mice ectopically expressing L1 in astrocytes (GFAP/L1 mice) [15] revealed an overexpression of 14-3-3β and ζ (T. Tilling et al., unpublished data).

The 14-3-3 family of protein-binding proteins was first discovered in brain, where it comprises ∼1% of total soluble protein [16]. 14-3-3 proteins are preferentially localized in neurons, but also expressed in a wide range of other cells and tissues [17]. The broad spectrum of 14-3-3 functions includes activation of tyrosine and tryptophan hydroxylases [18], regulation of the Raf-1 oncogene [19]–[21], and modulation of apoptosis [22], [23]. Consistent with their abundance in the brain, several studies point to an important role of 14-3-3 proteins in the nervous system. Genetic knock-out of 14-3-3 in *Drosophila* revealed an impairment of learning and synaptic plasticity [24]. In support of a similar function in mammals, Simsek-Duran et al. (2004) [25] have shown that 14-3-3 proteins are required for a presynaptic form of long-term potentiation in the mouse cerebellum. Moreover, members of the 14-3-3 family are involved in neuronal migration during vertebrate development [26], regulation of cerebellar NMDA receptor surface localization [27], and in neurotrophin-stimulated growth of neurites [28], [29].

The multitude of functions exerted by 14-3-3 proteins is achieved through their ability to bind to phosphoserine/phosphothreonine–containing motifs of their ligands in a sequence specific manner. Two of the best known 14-3-3 consensus binding motifs are RSXpSXP and RXXXpSXP (pS represents the phosphorylated serine residue) [30]. However, 14-3-3 proteins not only recognize these classical motifs, but also other phosphorylated sites and nonphosphorylated motifs [14], [31]. Owing to the versatility of binding sites in other proteins and to their ability to dimerize, 14-3-3 proteins act as adaptor proteins, chaperones and scaffolds [22].

For a better understanding of L1-mediated cell-cell interactions, it is crucial to elucidate the mechanisms of interaction between L1 and its intracellular binding partners [4], [5]. Based on the presence of a potential 14-3-3 binding motif in a functionally important part of the L1ICD, we posited that 14-3-3 proteins interact with the L1ICD and thereby play a role in L1 function. We show that 14-3-3 is indeed associated with L1 *in vivo*. Moreover, our results demonstrate that, on the one hand, CKII supports binding of 14-3-3 to L1 and, on the other hand, 14-3-3 binding to L1 enhances L1 phosphorylation by CKII. Finally, expression of the mutated form of 14-3-3ζ, which impairs interactions of 14-3-3 with its binding partners, stimulated neurite elongation on an L1 substrate. Taken together, our data indicate that 14-3-3 proteins regulate biological functions of L1.

## Results

### 14-3-3 is associated with L1

**Figure 1:**
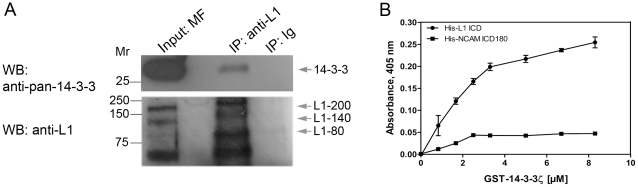
14-3-3 is associated with L1 *in vivo*. **A.** Immunoprecipitation (IP) of L1 from crude brain membrane fractions (MF) was performed using a rabbit polyclonal antibody to L1. Proteins were resolved by SDS-PAGE and analyzed by Western blotting (WB) with the anti-14-3-3 antibody H8. Successful immunoprecipitation of L1 was shown by Western blot analysis of the precipitates with a polyclonal anti-L1 antibody. The positions of full-length L1 (L1-200) and of proteolytic L1 fragments (L1-140 and L1-80; [69]) are indicated by grey arrows. **B.** **14-3-3ζ directly binds to the L1ICD.** Recombinantly expressed 6xHis-tagged L1ICD was immobilized on microtiter plate wells and assayed by ELISA for its ability to bind GST-14-3-3ζ. Measurement of the binding of 14-3-3ζ to the ICD of the unrelated neural cell adhesion molecule NCAM180 served as a negative control. Specific absorbance values were calculated by subtracting absorbance in wells incubated with GST only. Error bars denote standard deviation based on 3 independent experiments. Note that in some cases the error bars are not visible because of small standard deviations.

To investigate a possible association between L1 and 14-3-3 proteins in mouse brain, immunoprecipitation experiments were performed. L1 was immunoprecipitated from brain membrane fractions of 3-week-old mice using an L1- specific antibody. A non-immune rabbit IgG was used as a negative control. The presence of 14-3-3 proteins in the resulting anti-L1 immune complex was analyzed using an anti-pan-14-3-3 mouse monoclonal antibody. As shown in Fig. 1A, 14-3-3 co-immunoprecipitated with L1 from mouse brain membrane fractions, indicating that L1 and 14-3-3 proteins associate in the brain.

### 14-3-3ζ directly binds to the intracellular domain of L1

14-3-3ζ is one of the most abundantly expressed 14-3-3 isoform in neurons of the mammalian brain [32], [33]. Based on our finding that 14-3-3 proteins associate with L1, we hypothesized that 14-3-3ζ may bind to the L1ICD. To test this idea, an Enzyme-linked Immunosorbent Assay (ELISA)-based direct binding assay was performed. Recombinantly expressed L1ICD was immobilized on microtiter plate wells, and its ability to bind 14-3-3ζ was measured. Glutathione-S-transferase (GST)-14-3-3ζ bound in a concentration-dependent manner to the L1ICD (Fig. 1B), demonstrating that 14-3-3ζ directly binds to the L1ICD. There was no binding of GST-14-3-3ζ to the ICD of the 180 kDa isoform of the neural cell adhesion molecule NCAM (Fig. 1B), showing the specificity of the interaction between L1ICD and 14-3-3ζ.

### Ser^1181^ to Ala substitution and RSLESD deletion disrupt binding of L1 to 14-3-3ζ

**Figure 2:**
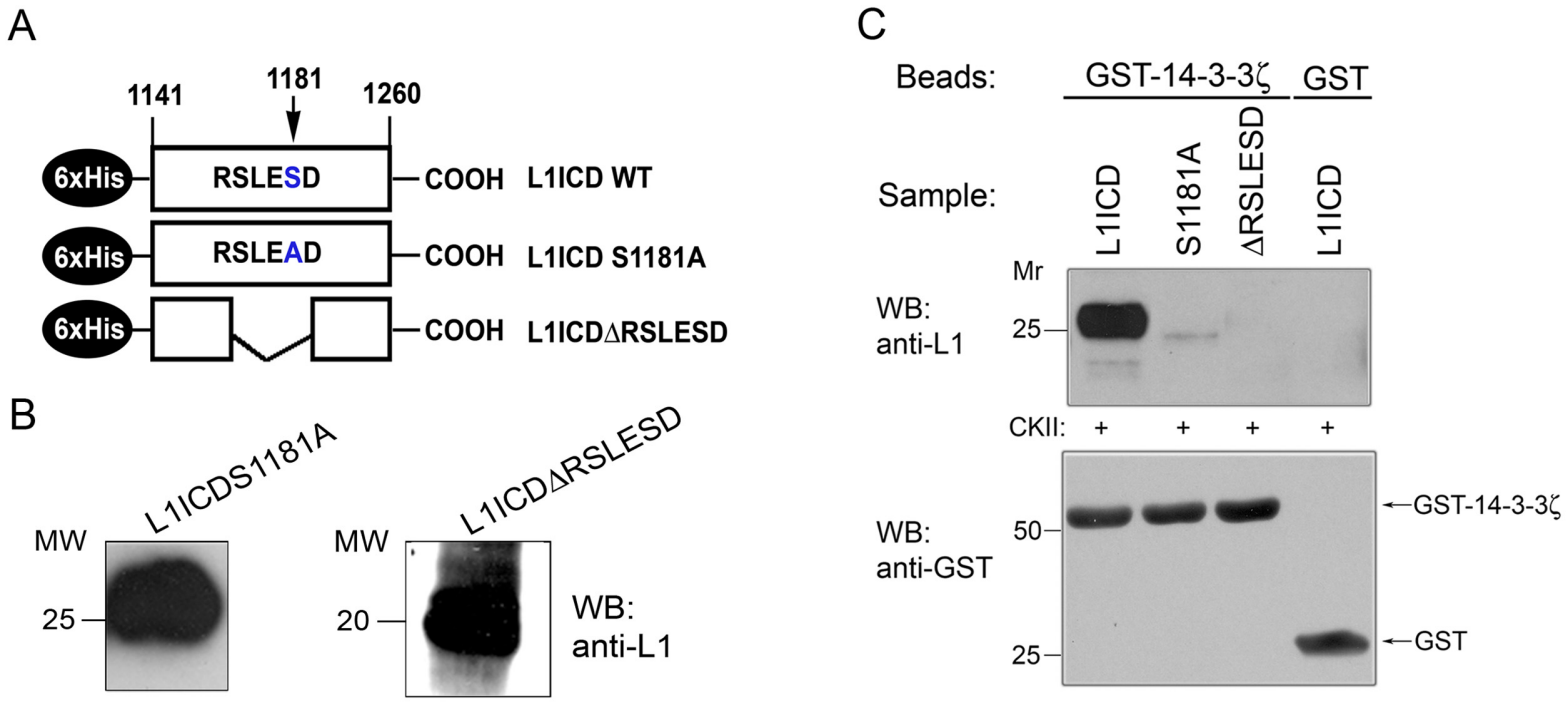
Ser^1181^Ala substitution and RSLESD deletion disrupt binding of L1 to 14-3-3ζ. **A.** Schematic of recombinant L1ICD constructs. The full-length L1ICD construct contains the RSLESD sequence, a potential 14-3-3 binding motif. L1ICD S1181A has a single amino acid substitution (S1181A) of a serine residue in this motif. Ser1181 can be phosphorylated by CKII. The RSLESD sequence, a potential 14-3-3-binding motif, is deleted in L1ICD ΔRSLESD. Wild-type and mutated L1ICD constructs were recombinantly expressed as 6xHis-tagged proteins, purified from bacterial lysates and used in pull-down experiments. **B.** Purified mutated L1ICD constructs were analyzed by Western blot using the 74-5H7 anti-L1 antibody. **C.** *Upper panel*: 6xHis-tagged proteins, purified from bacterial lysates, were subjected to GST-14-3-3ζ pull-down assays after treatment with CKII. GST was used as a control. Pull-down eluates were analyzed by Western blot (WB) with the 74-5H7 anti-L1 antibody. *Lower panel*, GST and GST-14-3-3ζ were detected in pull-down eluates by Western blot (WB) with an anti-GST antibody, confirming that comparable amounts of GST or GST-14-3-3ζ were used in each pull-down.

Next, we wanted to more closely define the 14-3-3 binding site in the L1ICD. The central part of the L1ICD contains the amino acid sequence RSLESD. The second serine within this sequence, Ser^1181^, can be phosphorylated by CKII [12] and RX_2-3_pS is a potential 14-3-3-binding motif [22]. Therefore, two L1ICD mutants were generated: L1ICD ΔRSLESD, in which the RSLESD had been removed, and L1ICD S1181A, in which Ser^1181^ was replaced by Ala to prevent L1 phosphorylation by CKII (Fig. 2A). Purified L1ICD mutants were analyzed by Western blot analysis with an anti-L1 antibody, which recognizes the unmutated form of L1ICD. As shown in Fig. 2B the L1 antibody also recognizes L1ICD S1181A and L1ICD ΔRSLESD. To assess whether 14-3-3 proteins bind the RSLEpSD sequence, we performed pulldown assays by comparing the ability of GST-tagged 14-3-3ζ to interact with *in vitro* CKII-phosphorylated L1ICD and the two mutants. While the unmutated L1ICD was specifically bound by 14-3-3ζ, the S1181A mutation strongly reduced and the RSLESD deletion abolished 14-3-3ζ binding (Fig. 2C, upper panel), indicating that 14-3-3 binding to L1 indeed requires the RSLESD sequence and that this binding is critically dependent on Ser^1181^. Comparable amounts of GST or GST-14-3-3ζ were used in each pull-down, as shown by Western blot analysis of pull-down eluates with a GST antibody (Fig. 2C, lower panel).

### 14-3-3ζ binds both to CKII phosphorylated L1ICD and to non-phosphorylated L1ICD

**Figure 3:**
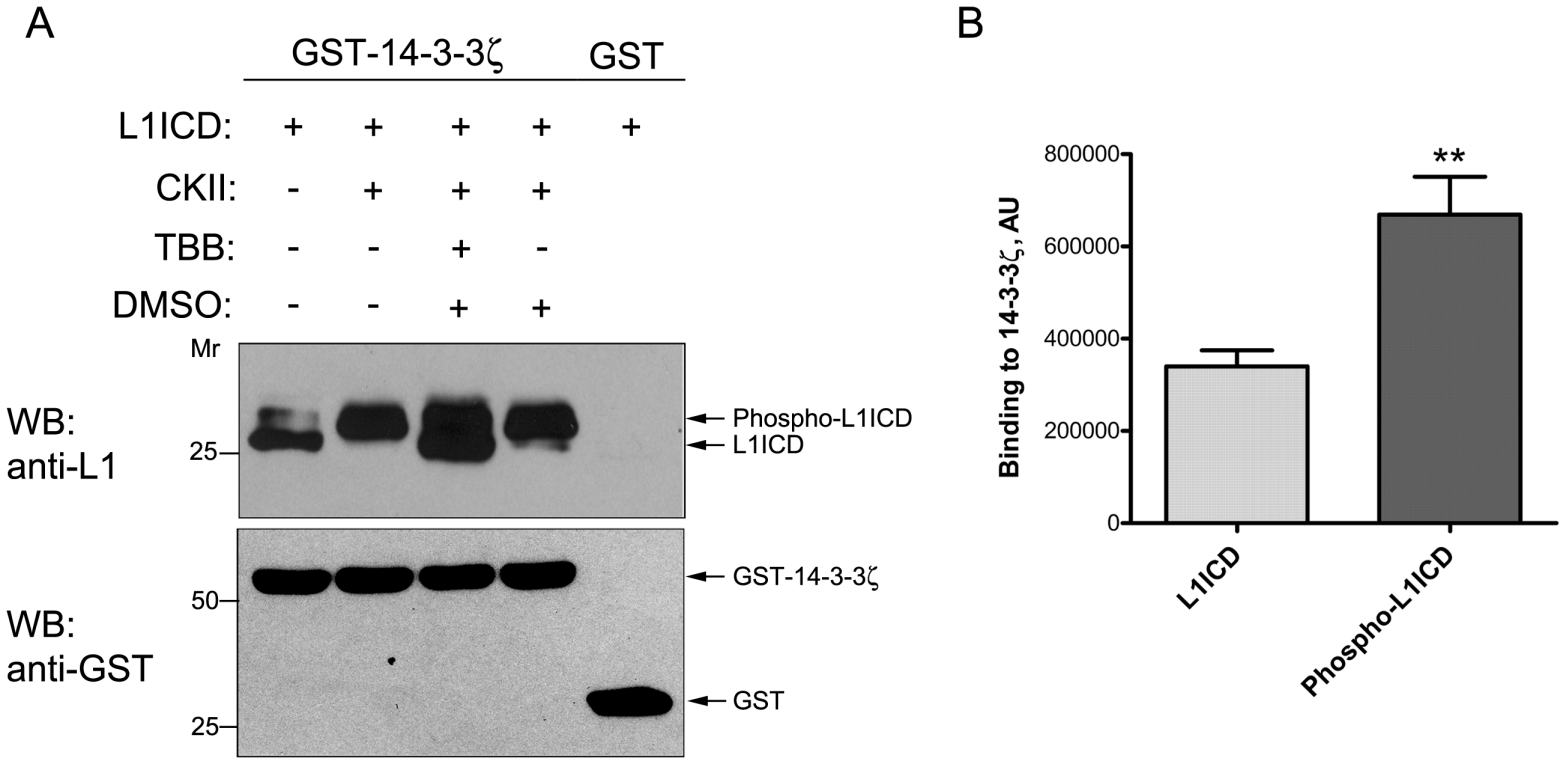
14-3-3ζ binds to CKII-phosphorylated L1ICD more strongly than to nonphosphorylated L1ICD. **A.** *Upper panel*: Equal amounts (15 µg each) of recombinant His-tagged L1ICD were incubated in the presence or absence of CKII. Where indicated, 5 µM TBB was included to specifically inhibit CKII. To exclude non-specific effects of DMSO in which TBB was dissolved, one reaction took place in the presence of this solvent. After treatment, a GST-14-3-3ζ pull-down was performed to investigate direct binding of L1ICD to 14-3-3. Pull-down eluates were analyzed by Western blotting (WB) with the 74-5H7 anti-L1 antibody. *Lower panel*, GST and GST-14-3-3ζ were detected in pull-down eluates by Western blotting (WB) with a GST antibody, confirming that comparable amounts of GST or GST-14-3-3ζ were used in each pull-down. **B.** CKII phosphorylation of L1ICD enhances its association with 14-3-3ζ. Quantification of the lower L1ICD-immunoreactive band in lane 1 (L1ICD only) and the upper L1ICD-immunoreactive band in lane 2 (L1ICD and CKII) was performed by densitometric analysis (**A**, *upper panel* shows a representative example). Data represent mean ±SEM of four independent experiments.

In the majority of cases documented so far, 14-3-3 interacts with phosphoproteins. However, our ELISA experiments showed that 14-3-3ζ is able to interact with nonphosphorylated recombinant L1ICD (cf. Fig. 1B). We therefore sought to more closely investigate whether phosphorylation of L1ICD by CKII at the Ser1181 residue affects its interaction with 14-3-3ζ. In order to monitor the specificity of CKII phosphorylation, 4,5,6,7- tetrabromobenzotriazole (TBB), a specific inhibitor of CKII [34], was utilized. We observed that 14-3-3ζ binds non-phosphorylated L1ICD (Fig. 3A, upper panel, lane 1) in line with our ELISA results, but also, even to a higher extent, phospho-L1ICD (Fig. 3A, upper panel, lane 2). A quantitative densitometric analysis confirmed that significantly more phospho-L1ICD bound to 14-3-3ζ compared to non-phosphorylated L1ICD (Fig. 3B), supporting a preferential interaction of 14-3-3ζ with CKII-phosphorylated L1. We also observed that inhibition of CKII activity by TBB led to the presence of non-phosphorylated L1ICD in the GST-14-3-3ζ eluate, as expected, although L1 phosphorylation was not completely blocked (Fig. 3A, upper panel, lane 3). Western blot analysis demonstrated that comparable amounts of GST and GST-14-3-3ζ were used in the pull-down assay (Fig. 3A, lower panel).

### 14-3-3ζ promotes CKII-catalyzed L1ICD phosphorylation

**Figure 4:**
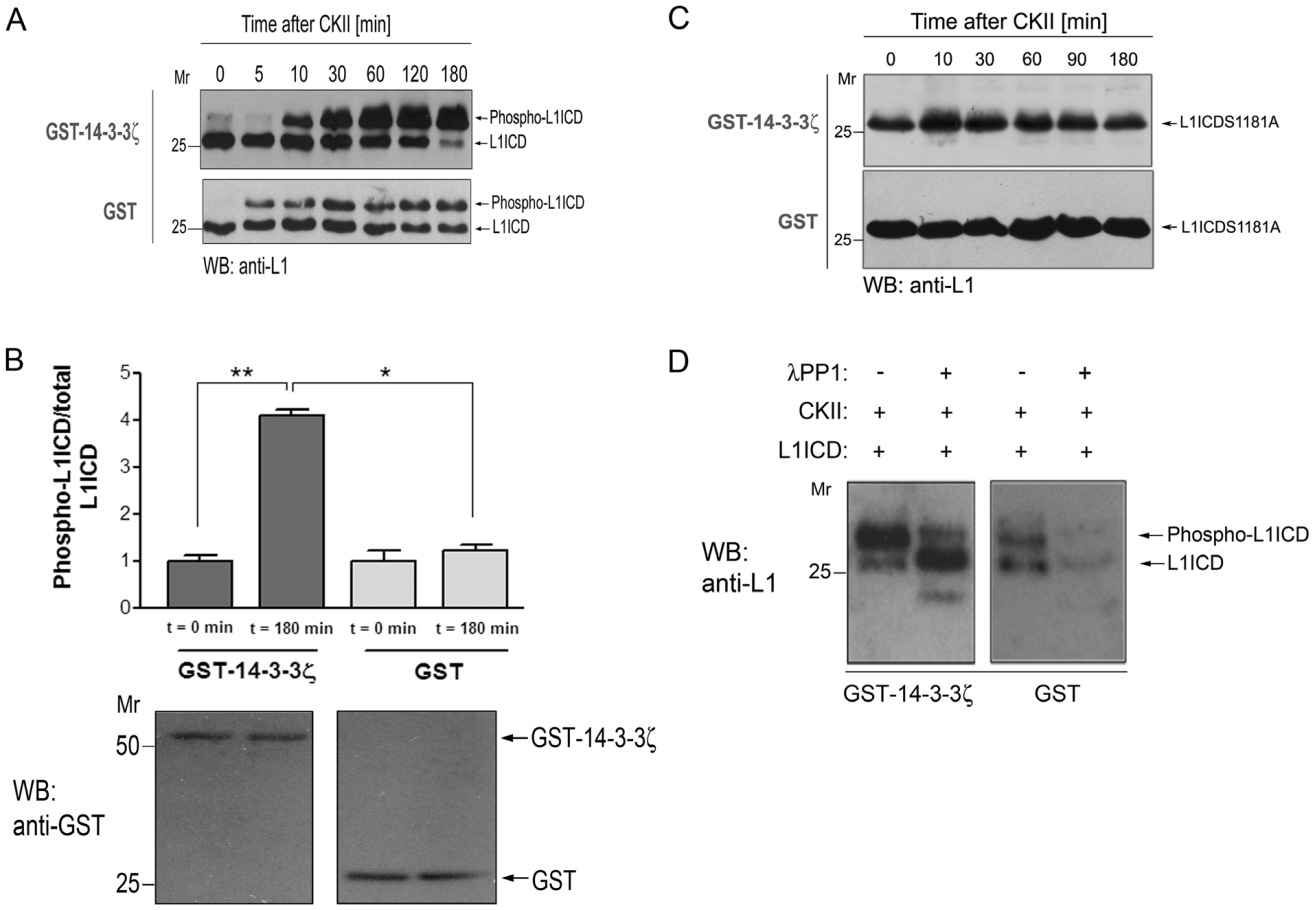
14-3-3ζ supports CKII-catalyzed L1ICD phosphorylation. **A.** L1ICD was preincubated in the presence or absence of GST-14-3-3ζ followed by incubation with CKII. At different time points, CKII phosphorylation was stopped by adding SDS loading buffer, and samples subjected to SDS-PAGE. Western blot analysis with the 74-5H7 anti-L1 antibody revealed that the band intensities of phosphorylated L1 (phospho-L1ICD) increased over time when L1ICD was preincubated with GST-14-3-3ζ (compare C). **B.** *Upper panel*, Comparison of L1ICD phosphorylation by CKII in the presence of GST-14-3-3ζ or GST. The phospho-L1ICD and L1ICD bands at time points 0 and 180 min (representative example given in panel **A**) were quantified and the ratio of phospho-L1ICD/total L1ICD at t = 0 min and t = 180 min in the presence of GST-14-3-3ζ or GST was calculated. Error bars denote ±SEM based on 3 independent experiments. *Lower panel*, GST Western Blot analysis of phosphorylation assay samples taken at the indicated time points confirms that equal amounts of GST or GST-14-3-3ζ were present in the reactions. **C.** L1ICD S1181A mutant was subjected to the same CKII phosphorylation assay as described in **A**. No band presumably representing newly phosphorylated L1ICD could be observed. **D.** Samples incubated with CKII for 180 min were subjected to subsequent treatment with λ protein phosphatase 1 (λPP1). Changes in the L1ICD band pattern were analyzed by Western Blot with the 74-5H7 antibody.

Having shown that CKII phosphorylation of the L1ICD is important for its interaction with 14-3-3ζ, we asked whether 14-3-3ζ binding to non-phosphorylated L1ICD influences CKII-catalyzed L1ICD phosphorylation. L1ICD was preincubated overnight with GST-14-3-3ζ (or GST only as a negative control) and then subjected to CKII phosphorylation. At serial time points, the reaction was stopped by adding SDS-PAGE loading buffer. The effect of 14-3-3ζ on L1ICD phosphorylation was analyzed by loading the samples directly onto an SDS gel and then performing Western blotting using an anti-L1 monoclonal antibody. Phosphorylation of L1ICD was evident by the appearance of a second protein band, presumed to be phosphorylated L1, with slightly lower mobility in SDS-PAGE relative to non-phosphorylated L1ICD (Fig. 4A). An increase in band intensities of phosphorylated L1 was observed over time (particularly from t = 0 min to t = 60 min) when L1ICD was preincubated with GST-14-3-3ζ (Fig. 4A). When L1ICD was preincubated with GST alone, there was a less pronounced increase in the respective band intensities from t = 0 min to t = 30 min. A densitometric comparison of band intensities at time points 0 and 180 min indicated that at the latter time point, L1ICD phosphorylation was ∼3 times stronger in the presence of 14-3-3ζ than in its absence (Fig. 4B). To confirm that the upper band represents phospho-L1ICD, the S1181A L1ICD mutant, in which Ser1181 is replaced by Ala to inhibit CKII–mediated L1 phosphorylation, was investigated in the same manner as wild-type L1ICD. In contrast to the non-mutated protein, no second band appeared over time with the S1181A mutant (Fig. 4C), demonstrating that the upper band observed in the experiments with wild-type L1ICD (cf. Fig. 4A) is indeed phosphorylated L1ICD and that the lower band represents non-phosphorylated L1ICD. To conclusively support this interpretation, we treated CKII-phosphorylated L1ICD with lambda protein phosphatase 1 (λPP1) (Fig. 4D). λPP1 treatment caused a shift towards the lower band, further confirming that the upper band represents phosphorylated L1ICD. In conclusion, these results indicate that 14-3-3ζ supports phosphorylation of L1ICD by CKII.

### 14-3-3ζ is associated with L1 in endosomes

**Figure 5:**
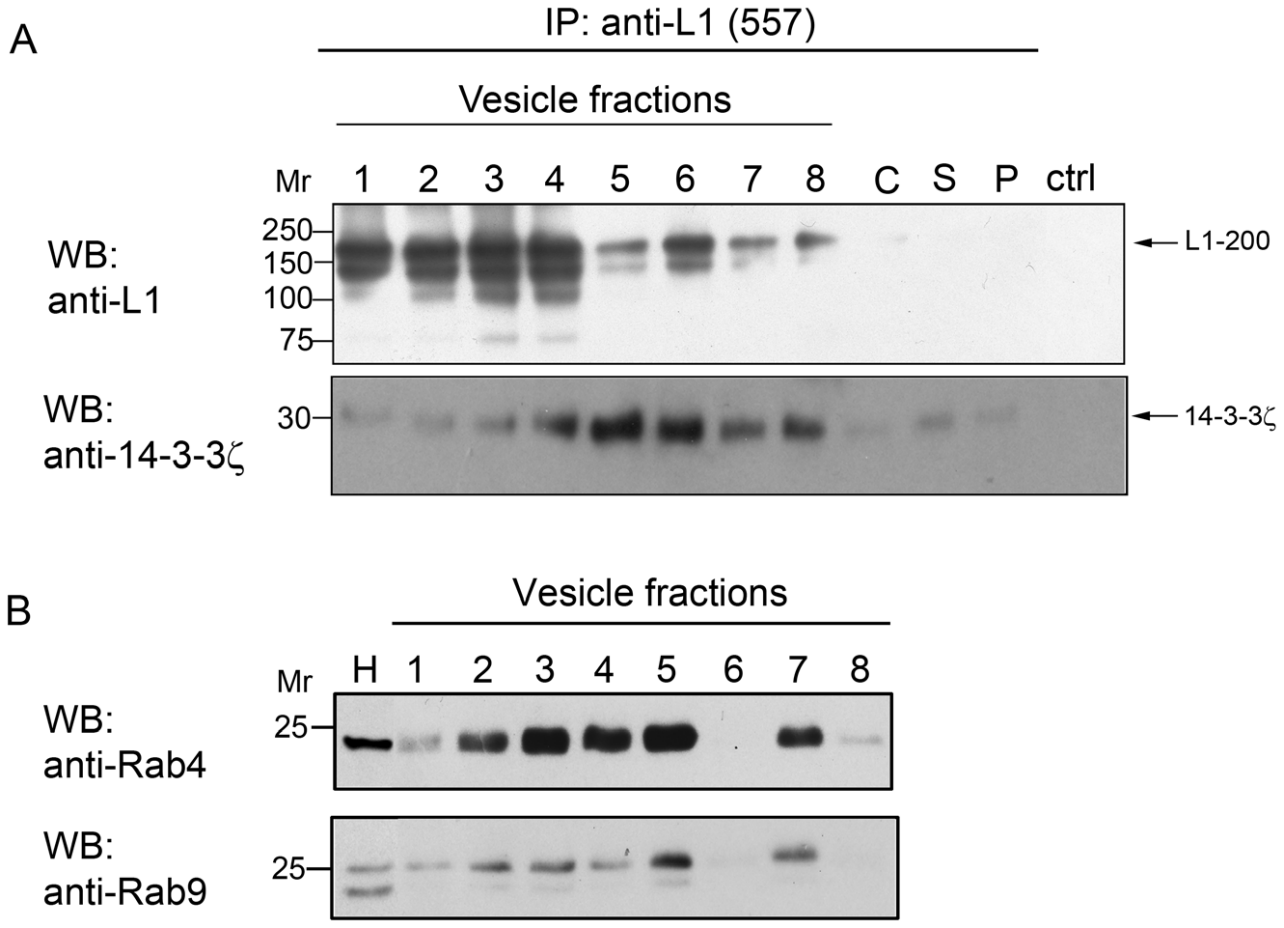
14-3-3ζ is associated with L1 in endosomes. **A.** *Upper panel*, Immunoprecipitation (IP) of L1 from vesicle fractions was performed using the anti-L1 monoclonal antibody 557. Proteins were resolved by a SDS-PAGE gradient gel (4–20%) and analyzed by Western blotting (WB) with an anti-L1 antibody (74-5H7), which recognizes full-length L1 and L1 proteolytic fragments containing the L1ICD. Full-length L1 (∼200 kDa), indicated by an arrow, was successfully precipitated. *Lower panel*, Western blot analysis of the L1 immunoprecipitates was also performed with an isoform-specific anti-14-3-3ζ antibody, revealing the association of 14-3-3ζ with L1 in vesicle fractions. C: crude endosomal preparation; S: cytosolic compounds; P: crude membrane fraction; ctrl: control IgG used for immunoprecipitation. **B.** To confirm that the isolated fractions are enriched in endosomal markers, equal protein amounts from total brain homogenate and vesicle fractions were analyzed for expression of the early endosome marker Rab 4 (upper panel) and the late endosome marker Rab 9 (lower panel). H: homogenate. In **B**, the bands shown for the homogenate are from a different blot, but with the same protein amount loaded and the same film exposure time as for the fractions.

CKII-mediated phosphorylation of L1 at Ser^1181^ has been implicated in normal endocytotic trafficking of L1 [13]. Recycling of endocytosed L1 occurs via sorting and recycling endosomes [35] and is important for L1-based growth cone motility [6]. We thus wished to analyze whether 14-3-3ζ is associated with L1 in endosome-enriched vesicle fractions. The enrichment of certain types of vesicles in the different fractions can be analyzed by determination of known vesicular resident components such as the Rab small GTPases [36], [37]. Membrane preparations from postnatal day 7 C57BL/6J mouse brains were subfractionated utilizing a sucrose gradient to obtain vesicle fractions. From these fractions, L1 was precipitated using the anti-L1 monoclonal antibody 557. Western blot analysis of the L1 immunoprecipitates showed that full length L1 (∼200 kDa) and proteolytically cleaved L1 (lower bands) were successfully precipitated, with noticeable enrichment in fractions 3, 4 and 8 (Fig. 5A, top panel). Detection with an isoform-specific anti-14-3-3ζ antibody revealed the presence, albeit at varying amounts, of 14-3-3ζ (∼30 kDa) in L1 immunoprecipitates (Fig. 5A, bottom panel), suggesting that 14-3-3ζ is associated with L1 in vesicles. Interestingly, we observed that the highest amount of 14-3-3 was associated with L1 in endosomal fraction 5, which contains relatively low amounts of L1 (Fig. 5A). In order to characterize these fractions more precisely, we performed Western blot analyses using antibodies against Rab marker proteins specifically expressed in two distinct endosome populations. Both early (Rab 4; [38]) and late endosome markers (Rab 9; [39], [40]) were detected in fractions 2-5 and 7. Substantially lower amounts of these two markers were detected in fractions 1 and 8 and none of the two markers was present in fraction 6 (Fig. 5B). These results suggest that 14-3-3 is associated with L1 in particular types of endosomes and support an involvement of 14-3-3 in L1 sorting and trafficking.

### Expression of 14-3-3ζ K49E leads to an increase of neurite length in L1-mediated neurite outgrowth

**Figure 6:**
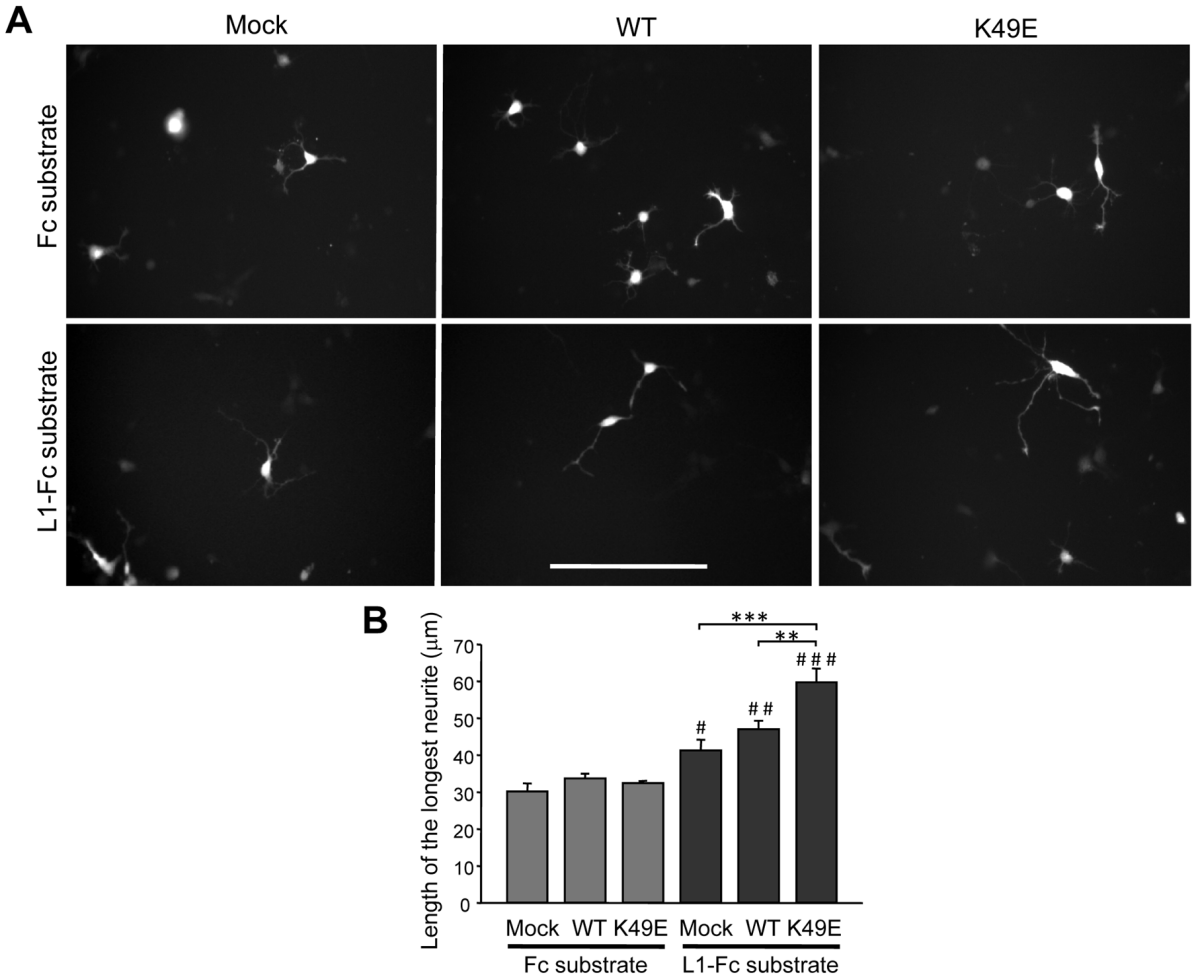
Expression of 14-3-3ζ K49E leads to a specific increase in L1-mediated neurite outgrowth. Hippocampal neurons prepared from embryonic rat hippocampus were transfected by nucleofection with an expression plasmid for EGFP together with plasmids encoding either empty vector (“Mock”), wild-type 14-3-3ζ (“WT”) or 14-3-3ζ K49E (“K49E”). Subsequently, cells from each transfection were plated onto wells coated with PLL in combination with Fc (“Fc substrate”) or L1-Fc (“L1-Fc substrate”). After incubation for 24 h at 37°C, cells were fixed. **A.** The images show transfected neurons in three transfected groups grown either on Fc or L1-Fc substrates. **B.** Length of the longest neurite per cell was measured for neurons in the same groups as in (**A**.). Values are expressed as mean ±SEM. Per substrate and expressed protein, neurons from 3 transfections, 3 wells per transfection, ∼30 cells per well were analyzed. Two-way ANOVA with repeated measures (for substrate) revealed significant effects of transfection (p<0.05), substrate (p<0.001) and interaction between transfection and substrate (p<0.05). **p<0.01, ***p<0.001, statistically significant differences vs. 14-3-3 K49E; #p<0.05, ##p<0.01, ###p<0.001, statistically significant differences between neurons grown on Fc and L1-Fc (Bonferroni's post-hoc test, n = 3 transfections).

Our above-mentioned observations suggested a role for 14-3-3 in regulating L1 phosphorylation by CKII, putatively in endosomes. Considering the importance of endosomal trafficking for L1-mediated neurite growth [6] and the suggested involvement of CKII in this process [12], we wanted to examine whether 14-3-3 influences L1-mediated neurite outgrowth. For this purpose, either wild-type 14-3-3ζ or a K49E mutated form of 14-3-3ζ was overexpressed in hippocampal neurons plated on L1-Fc or on Fc control substrate. The K49E mutation replaces a crucial Lys residue found in the amphipathic binding groove of all 14-3-3 isoforms by Glu, thus, abolishing most, if not all, interactions of 14-3-3 with its binding partners [41]. We observed that the total length of neurites grown on the L1-Fc substrate was significantly increased in 14-3-3ζ K49E expressing cells relative to wild-type 14-3-3ζ- and mock-transfected control cells, whereas no differences were observed between mock- and wild-type 14-3-3ζ-transfected cells (Fig. 6). Given that no significant difference was observed for mock, 14-3-3ζ wild-type and K49E transfected neurons grown on the Fc substrate (Fig. 6), we conclude that the 14-3-3ζ K49E enhanced stimulation of neurite elongation is L1-specific.

## Discussion

In the present study 14-3-3 and L1 were found to associate *in vivo*, as demonstrated by coimmunoprecipitation. ELISA and pull-down experiments confirmed a direct interaction between 14-3-3ζ and the intracellular domain of L1 (L1ICD). We have also observed binding of recombinant 14-3-3β to L1ICD *in vitro* (E.M. Ramser et al., unpublished observations). However, the binding intensity was lower, suggesting that L1 may preferentially interact with specific 14-3-3 molecules. In the present study, we focused on the interaction between 14-3-3ζ and L1ICD. This interaction is enhanced by CKII-mediated phosphorylation of L1ICD. Notably, we found that 14-3-3ζ interacts not only with phosphorylated L1, but also with nonphosphorylated L1. Although most known 14-3-3-ligands possess phosphoserine- or phosphothreonine-based motifs, several interactions between 14-3-3 and nonphosphorylated motifs within ligand proteins have been described. Examples include the sequences VTPEER of the amyloid protein precursor (APP) ICD fragment [42], and WLDLE of the synthetic peptide R18 [43], [44]. It is generally accepted that phosphorylation-independent interactions between 14-3-3 and its binding partners occur in a manner similar to that of the binding partner pS-RAF. This assumption is based on the finding that R18 was co-crystallized with 14-3-3 in a position similar to that of phosphorylated pS-RAF-259 [43]. In the complex structure of 14-3-3ζ with R18, the aspartate (D) and glutamate (E) side chains generated a negative charge density comparable to that of phosphoserine of pS-RAF-259 [43].

The RSLESD sequence in the central part of the L1ICD, which, as discussed below, is the likely site of interaction for 14-3-3ζ, contains two acidic residues. These residues might mimic a phosphoryl group of consensus motifs and help to mediate the interaction of 14-3-3ζ with non-phosphorylated L1. Mutation of these two residues may be a suitable approach to clarify the mechanism of phospho-independent 14-3-3 binding to L1 in the future. The RSLESD sequence contains a RX2-3pS motif, which is a potential 14-3-3-binding site [22]. We therefore focused on this sequence for further analysis, particularly the second Ser residue that is known to be phosphorylated by CKII [12]. This CKII phosphorylation site is evolutionarily well-conserved among L1 orthologs and L1 family CAMs [45] and, therefore, is likely to be required for subsequent interactions of L1 proteins in signaling cascades and to serve a significant role in L1 function. Mutation analysis showed that the RSLESD sequence is indeed the site of 14-3-3 interaction and confirmed that phosphorylation by CKII occurs at Ser^1181^. Moreover, we found that Ser^1181^ is indispensable for the interaction of 14-3-3ζ with L1, as 14-3-3 failed to bind an S1181A L1ICD mutant in pull-down assays. This finding suggests that Ser^1181^ is also crucial for the interaction between 14-3-3 proteins and non-CKII-phosphorylated L1. At present we cannot completely exclude that additional phosphorylated or non-phosphorylated 14-3-3 binding sites exist in the L1ICD. Based on the “gatekeeper phosphorylation” concept [46], one could speculate that high-affinity binding of one monomeric subunit of a 14-3-3ζ dimer to the RSLESD motif in L1 could be the prerequisite for binding of a second 14-3-3 monomeric subunit to a second, lower-affinity site in L1. However, our mutagenesis data strongly indicate that the RSLESD motif identified in this study is the principal site of interaction with 14-3-3ζ. Moreover, the “gatekeeper concept” is based on phosphorylation of the primary interaction site, whereas our data show that 14-3-3ζ also interacts with non-phosphorylated L1.

As 14-3-3ζ interacts with nonphosphorylated and CKII-phosphorylated L1ICD, the question arose whether 14-3-3ζ utilizes the identified binding site (RSLESD) for phosphorylation-dependent and –independent interaction with L1. Notably, two previous studies showed that 14-3-3ζ interacts with phosphorylated and non-phosphorylated Tau protein and that two distinct 14-3-3 binding sites mediate these interactions [47], [48]. Both of these studies also showed that phosphorylation of the Tau protein by PKA increased its affinity for 14-3-3ζ. In line with this finding, we observed that CKII phosphorylation enhances binding of 14-3-3ζ to L1ICD. These observations suggest a two-step mechanism for the 14-3-3 – L1 interaction: in a first step, 14-3-3 binds to its non-phosphorylated L1 with relatively low affinity. Subsequent serine phosphorylation of L1, e.g. as a consequence of a physiological stimulus, then tightens the 14-3-3 – L1 interaction. Phosphorylation of tyrosine 1176 (Tyr^1176^), a residue adjacent to the RSLESD sequence essential for 14-3-3 binding, has been shown to prevent binding of L1 to the AP-2 adaptor protein, thereby blocking L1 endocytosis [49]. Initial binding of 14-3-3 to L1 prior to CKII phosphorylation might thus also be controlled by the phosphorylation status of Tyr^1176^, which could act as a regulatory signal. However, binding of L1 to CKII-phosphorylated 14-3-3ζ *in vitro* is not altered in the presence of AP-2 (E.M. Ramser, unpublished observations).

The above-mentioned study on 14-3-3 - Tau interaction [47] showed that association of 14-3-3 with the microtubule binding region of Tau stimulates phosphorylation of serine residues within this region. We thus investigated whether 14-3-3 proteins have a similar effect on CKII-mediated L1 phosphorylation. Notably, phosphorylation of L1ICD at Ser^1181^ by CKII was profoundly enhanced by 14-3-3ζ. One explanation is that L1 phosphorylation is promoted by initial interactions between 14-3-3ζ and (non-phosphorylated) L1ICD, resulting in L1ICD conformational changes and, consequently, increased susceptibility for CKII phosphorylation. It is known that 14-3-3 behaves like a molecular anvil, deforming its bound ligands while undergoing only minimal structural alterations itself [46]. For example, in the case of serotonin N-acetyl transferase, and presumably also exoenzyme S, 14-3-3 binding deforms the catalytic residues, resulting in enhanced substrate binding and product formation [50], [51]. For other proteins, 14-3-3-mediated conformational changes might facilitate the interaction with their binding partners, leading for example to enhanced phosphorylation [46]. Another possible explanation for 14-3-3-enhanced phosphorylation of L1 by CKII is that 14-3-3ζ acts as a scaffolding protein by recruiting CKII to L1ICD. Even though there is no direct evidence at present for an association between 14-3-3ζ and CKII, other studies have shown that 14-3-3ζ forms homo- or heterodimers, a prerequisite for acting as a scaffolding protein [42], [52]. Therefore, further experiments investigating the CKII-catalyzed phosphorylation of L1 in the presence of a 14-3-3ζ dimer and a dimerization-deficient 14-3-3ζ mutant could help to further elucidate how the dimeric structure of 14-3-3ζ may influence L1 phosphorylation. Finally, considering the time course of CKII-mediated L1 phosphorylation in our study, it is plausible to assume that 14-3-3 stabilizes the CKII-phosphorylated form of L1. Dent et al. showed that 14-3-3-bound proteins are resistant to phosphoprotein phosphatases [53]. In our case, binding of 14-3-3 to L1 might thus protect pSer1181 from dephosphorylation.

Nakata and Kamiguchi [13] have suggested that CKII regulates endocytotic L1 trafficking in the axonal growth cone via phosphorylation of the L1ICD. Considering our data on L1, 14-3-3ζ and CKII, 14-3-3ζ might play a role in controlling L1 sorting and trafficking between endosomes and plasma membranes. We therefore investigated whether L1 and 14-3-3ζ interact in vesicle fractions from mouse brain. We could show that 14-3-3ζ associates with L1 in early and late endosome-enriched fractions, which further supports an involvement of 14-3-3 proteins in L1 sorting and trafficking.

Given the importance of L1 trafficking for neurite extension, we also tested the influence of neuronally expressed 14-3-3ζ [17] on L1-mediated neurite growth. We observed that expression of 14-3-3ζ K49E in hippocampal neurons led to an increase in neurite elongation on an L1 substrate. Only cells grown on an L1-Fc substrate, but not on Fc responded to 14-3-3ζ K49E expression, suggesting that 14-3-3ζ is a specific regulator of L1-dependent neurite extension. This effect depended on the amphipathic groove shared by all 14-3-3 isoforms [41], as the K49E mutant of 14-3-3 worked as a dominant-negative protein promoting L1-triggered neurite outgrowth, presumably by competing with the 14-3-3ζ wild-type form in the growth cones. The K49E mutation abolishes binding of 14-3-3ζ to various ligands, as has been shown in several studies [41], [54]–[56]. As an alternative approach, it would be possible to knockdown 14-3-3ζ by RNA interference and test the effect of this knockdown on L1-mediated neurite outgrowth. However, given the high degree of conservation between 14-3-3 isoforms, other isoforms than zeta are likely to compensate for its function in such a situation. We therefore opted for the dominant-negative approach.

Based on our biochemical data, it is intriguing to speculate that expression of dominant-negative 14-3-3ζ K49E causes a decrease in L1 phosphorylation by CKII on Ser^1181^. This, in turn, could inhibit targeting of L1 to late endosomes and lysosomes, resulting in an increased amount of L1 molecules on the cell surface. If more L1 molecules are available on the cell surface, this will stimulate the neuron's homophilic response to an L1 substrate, in accordance with our results. In line with such a model, an involvement of 14-3-3 proteins in the regulation of the subcellular localization of target proteins and the stabilization of targets at the cell surface has been demonstrated by several studies (e.g. [57], [58]; see [59], [60] for reviews). In this context, one might argue that 14-3-3 could also act on molecules downstream of L1, like the p90 ribosomal S6 kinase 1 (p90rsk). p90rsk is bound by 14-3-3, which negatively regulates its activity [61]. As p90rsk-mediated phosphorylation of L1 appears to be involved in L1-dependent neurite growth [62], an increased p90rsk activity caused by 14-3-3ζ K49E expression might also promote neurite extension on L1.

Summarizing, our study not only characterizes a novel interaction between a neural cell adhesion molecule L1 and the family of 14-3-3 intracellular signaling proteins, but also demonstrates the importance of this interaction for CKII-mediated phosphorylation of L1. Moreover, our cell culture experiments identify 14-3-3ζ as a putative modulator of L1-dependent neurite growth. These findings contribute to a better understanding of the molecular mechanisms underlying the crucial role L1 plays in nervous system development, regeneration and plasticity.

## Materials and Methods

### Ethics statement

All animal experiments were conducted in accordance with the Italian and European Community laws on protection of experimental animals, and the procedures used were approved by the Office of Animal Welfare at the Department of Veterinary Public Health, Nutrition and Food Safety in Rome (permit number: 223).

### Constructs and mutagenesis

pDEST15-14-3-3ζ, pDEST26-14-3-3ζ, and pDEST26-14-3-3ζ K49E were kindly provided by Dr. H. Fu, Emory University, Atlanta. pDEST15-14-3-3ζ was used for expression of GST-14-3-3ζ in *E. coli*. pDEST26-14-3-3ζ and pDEST26-14-3-3ζ K49E were transfected into mammalian cells for recombinant expression of His-14-3-3ζ or His-14-3-3ζ K49E, respectively. To enable prokaryotic expression of His-tagged L1ICD, including the amino acids Cys-Phe-Ile (CFI) of the transmembrane domain, the following steps were performed: RNA from murine brain was extracted with TRIzol according to the manufacturer's manual (Invitrogen, Karlsruhe, Germany). The QuantiTect Rev. Transcription Kit was used for cDNA synthesis according to the manufacturer's protocol (Qiagen, Hilden, Germany). L1 CFI was amplified from cDNA using gene specific primers and AccuPrime™ *Taq* DNA Polymerase (Invitrogen). The polymerase chain reaction (PCR) was started with 2 min at 94°C and followed by 30 cycles of 30 sec at 94°C, 30 sec at 55°C, and 60 sec at 68°C. All primers were synthesized by Metabion (Martinsried, Germany) based on the L1cam DNA sequence (NCBI accession number NM_008478.3). The PCR product was first ligated into pGEM-T Easy (Promega, Mannheim, Germany) using TA cloning, and subsequently subcloned into the expression vector pQE30 (Qiagen) using *Bam*H I and *Hin*d III. Plasmids containing single point mutations and the RSLESD deletion construct were prepared using the QuikChange II XL Site-Directed Mutagenesis Kit (Stratagene, Amsterdam, The Netherlands). All constructs were verified by DNA sequencing.

### Antibodies

Anti-14-3-3 (sc-1657), which recognizes all 14-3-3 isoforms according to the supplier's information, and anti-Rab 9 (sc-28573) antibodies were purchased from Santa Cruz Biotechnology (Heidelberg, Germany), anti-14-3-3ζ (JP 18644) from IBL (Hamburg, Germany), anti-Rab 4 (610888) antibody from BD Transduction Laboratories (Heidelberg, Germany) and the anti-GST antibody from GE Healthcare (Freiburg, Germany). Polyclonal antibodies against the mouse L1 extracellular domain were obtained by immunizing rabbits with a protein A-purified L1-Fc fusion protein consisting of the extracellular domain of mouse L1 and the Fc portion of human IgG [63]. The rat monoclonal against anti-mouse L1 antibody 557 [64] recognizes an epitope localized in the extracellular domain of L1. The anti-L1 mouse monoclonal antibody 74-5H7 [9], which recognizes an epitope in the intracellular domain of L1, was purchased from HISS Diagnostics (Freiburg, Germany).

### Proteins

GST-14-3-3ζ fusion protein was expressed in *E. coli* BL21-AITM cells (Invitrogen) by L-arabinose induction. His-L1ICD, His-L1ICDS1181A, and His-L1ICDΔRSLESD were expressed in *E. coli* M15 cells by isopropyl-β-D-thiogalactoside induction and purified on Ni-NTA-agarose (Qiagen) and Ni-TED-agarose (Macherey-Nagel, Düren, Germany) according to the manufacturer's instructions. His-NCAM180 ICD [65] was provided by D. Novak from our laboratory. L1-Fc [63] was provided by G. Loers from our laboratory, and used for coating of wells in neurite outgrowth experiments. Poly-l-lysine (PLL) was purchased from Sigma (Taufkirchen, Germany), human Fc was from Dianova (Hamburg, Germany).

### Sucrose density gradient fractionation

Brains from 7-day-old C57BL/6J mice were homogenized in homogenization buffer (0.32 M sucrose, 50 mM Tris-HCl (pH 7.4), 1 mM CaCl_2_, and 1 mM MgCl_2_, 1 mM phenylmethylsulfonyl fluoride (PMSF), Complete Protease Inhibitor Cocktail, EDTA-free (Roche Diagnostics, Mannheim, Germany), and Phosphatase Inhibitor Cocktail I (Sigma)). Brain homogenates were centrifuged at 17,000×g for 1 h. The 17,000×g supernatant thus obtained was centrifuged at 100,000×g for 1 h. The subsequent 100,000×g pellet was homogenized in 0.32 M sucrose in Tris buffer (50 mM Tris-HCl (pH 7.5), 1 mM CaCl_2_, and 1 mM MgCl_2_) and loaded onto a step gradient comprising layers of 2, 1.3, 1.1, 0.8, 0.5 and 0.25 M sucrose as described [66], [67]. Gradients were centrifuged at 100,000×g for 2 h. Eight 1 ml fractions were collected from the top of the gradient and homogenized in 0.32 M sucrose in Tris buffer. After another centrifugation step at 100,000×g for 30 min, each of the eight fractions, enriched in distinct endosomes, was collected. These vesicle fractions were used for immunoprecipitation assays and Western blot analysis with the indicated antibodies.

### Enzyme Linked Immunosorbent Assay (ELISA)

Intracellular domains of L1 and NCAM180 (5 µg/ml) were immobilized overnight on a polyvinyl chloride surface (Nunc, Roskilde, Denmark) in TBS (10 mM Tris-HCl pH 7.4 and 150 mM NaCl). Wells were then blocked for 1 h with TBS containing 1% BSA and incubated for 1 h at RT with increasing concentrations of GST-14-3-3ζ (or GST-only control) diluted in buffer A (1% BSA, 1 mM CaCl_2_, 1 mM MgCl_2_ in TBS-Tween (0.05% Tween)). Plates were washed 3 times with TBS-T and incubated for 1 h with anti-GST goat polyclonal antibody diluted 1∶4000 in TBST containing 1% BSA. After washing with TBST, wells were incubated with peroxidase-coupled secondary antibody in TBS-T containing 1% BSA, washed 4 times, and incubated with 0.1% ABTS substrate (Roche Diagnostics) in 100 mM acetate buffer, pH 5.0. Absorbances were measured at 405 nm. Correction for potential GST background signals was performed by subtracting absorbance values in wells incubated with GST only.

### *In vitro* phosphorylation assay

CKII mediated phosphorylation was investigated by incubating 15 µg of L1ICD proteins at 30°C for 3 h in a reaction mixture containing 20 mM Tris- HCl (pH 7.5), 50 mM KCl, 10 mM MgCl_2_, 1 mM dithiothreitol (DTT), and 200 µM ATP with 2000 units/mL of CKII (New England Biolabs, Schwalbach, Germany). The same assay was also performed with L1ICD proteins preincubated with GST or GST-14-3-3ζ at 4°C for 16 h. Where indicated, λ protein phosphatase 1 (λPP1, New England Biolabs) was added to aliquots of the CKII reaction mixture after 3 h, yielding a λPP1 concentration of 6000 units/mL. These aliquots were supplemented with 50 mM HEPES (pH 7.5), 100 mM NaCl, 1 mM MnCl_2_, 2 mM DTT and 0.01% Brij 35, and incubated for 1 h at 30°C. To determine L1ICD phosphorylation, reaction aliquots were withdrawn at the indicated time points, mixed with an equal volume of SDS-PAGE sample buffer, and analyzed by SDS-PAGE and Western blotting. Blots were scanned and band intensities were determined.

### GST Pull-down assay

20 µg GST-14-3-3ζ fusion protein or an approximately equimolar amount of GST (10 µg) were coupled to 50 µl Glutathione Sepharose 4B beads (Sigma) for 2 h at 4°C, followed by preincubation with 5% BSA in pull-down buffer (20 mM Tris-HCl pH 7.4, 300 mM NaCl, and 0.05% Nonidet P-40) for 2 h at 4°C. Beads were washed with pull-down buffer, and incubated with the total CKII phosphorylation reaction described above. Agarose beads were collected by centrifugation and washed extensively with 0.1% Nonidet P-40 pull-down buffer. Samples were analyzed by SDS-PAGE and Western blotting.

### Co-Immunoprecipitation (Co-IP) of L1 and 14-3-3

Mouse brain membrane fractions containing 1.5 mg/ml protein in modified RIPA buffer (50 mM Tris-HCl pH 7.4, 150 mM NaCl, 2 mM EDTA, 1 mM NaF, 1 mM Na_3_VO_4_, 1% Nonidet P-40, 0.5% SDS, 100 µM PMSF, Complete Protease Inhibitor Cocktail EDTAfree, and Phosphatase Inhibitor Cocktail I) were precleared using 25 µl of Protein A Agarose beads (Santa Cruz). Immunoprecipitation was performed with an anti-mouse L1 polyclonal antibody and as a negative control an nonimmune rabbit IgG. After 4 h of incubation, 25 µl of Protein A Agarose beads were added to the supernatant, and incubation continued overnight at 4°C. Protein A Agarose beads were collected by centrifugation, and washed 4 times with RIPA buffer. Bound proteins were eluted by boiling in SDS-PAGE sample buffer and subsequently analyzed by Western blotting using the indicated antibodies. Mouse brain vesicle fractions were lysed in lysis buffer (50 mM Tris-HCl, pH 8.0, 1 mM EDTA, 150 mM NaCl, 1% Nonidet P-40, 1 mM Na_4_P_2_O_7_, 1 mM NaF, 2 mM Na_3_VO_4_, and Protease Inhibitor Cocktail, EDTA-free) for 1 h at 4°C. Extracts were centrifuged at 21,000×g for 30 min at 4°C and the supernatants were further analyzed. Vesicle fractions containing 200 µg/ml protein in Protein A/G binding buffer (Pierce, Bonn, Germany) were precleared using 20 µl Protein G magnetic beads (Thermo Scientific) for 30 min at 4°C. Immunoprecipitation was performed with 5 µg anti-L1 antibody 557 and purified non-immune rat IgG as a control overnight at 4°C. Antibody-protein complexes were precipitated using 20 µl of Protein G magnetic beads. Samples were analyzed by SDS-PAGE and Western blotting.

### Culture of primary hippocampal neurons

A protocol for preparation, culture and transfection of primary rat embryonic E18 hippocampal neurons and determination of neurite length was adapted from [68]. Cell were co-transfected with 0.3 µg pmaxGFP® (Amaxa, Cologne, Germany) and 0.3 µg DNA of either 14-3-3ζ or 14-3-3ζ K49E or an empty vector (pcDNA3) using the Amaxa® basic neuron small cell number nucleofection kit, Nucleofector® II and program 1 for small cell number. 300,000 cells were used per transfection and 12,000 were seeded per well in 96-well plates (BD Biosciences, Heidelberg, Germany) with thin glass bottom consequently coated with PLL (100 µg; Sigma) and 37.5 nM of either Fc or L1-Fc. The culture medium contained Neurobasal A medium supplemented with 2% B27 (both from Invitrogen). For neurite length measurements, digital images were automatically acquired with BD Pathway Imager using a 20× objective. The length of the longest neurite of enhanced green fluorescent protein (EGFP)-positive cells was measured by a trained operator using ImageJ software (http://rsb.info.nih.gov/ij/) without knowledge of substrate and vectors used for transfection. Statistical analyses were performed by Bonferroni's post-hoc comparison after two-way ANOVA with repeated measures (SigmaStat 3.5).
